# Comparing Two Vancomycin Loading Dose Regimens in Patients With Obesity: A Single-Center Prospective Clinical Trial

**DOI:** 10.7759/cureus.70849

**Published:** 2024-10-04

**Authors:** William Payne, Stephanie Thompson, Brian N Burton

**Affiliations:** 1 Emergency Medicine, Charleston Area Medical Center, Charleston, USA; 2 Research, Charleston Area Medical Center, Charleston, USA

**Keywords:** antibacterial agent, critical ill patients, nephrotoxicity, obesity, pharmacokinetics, prospective study, randomized controlled trial, renal function, vancomycin

## Abstract

Background: Obesity can significantly influence the pharmacokinetics of several medications, including vancomycin. Consequently, specialized dosing strategies may be required to ensure efficacy and safety in patients with obesity (PwO) requiring treatment with vancomycin. This single-center, prospective study evaluated two vancomycin loading dose regimens in PwO, comparing serum vancomycin concentrations, adverse events, and the impact on renal function.

Methods: Adult patients weighing over 100 kg were randomized into two study groups. A 20 mg/kg loading dose of vancomycin was administered to both groups, with one group restricted to a maximum dose of 2000 mg (n=33) and the other group receiving up to 4000 mg (n=34).

Results: Patients receiving the 4000 mg maximal dose achieved significantly higher median vancomycin concentrations at the initial trough (9.1 mg/L vs. 11.3 mg/L, p=0.0497), mean concentrations at the 7.5-hour interval trough (14.4 mg/L vs. 17.1 mg/L, p=0.0151), and mean concentrations at the post-load peak (23.9 mg/L vs. 30.9 mg/L, p=0.0023), without a corresponding increase in adverse renal outcomes, hospital length of stay, or mortality as compared with the 2000 mg maximum dose group.

Conclusions: The study’s findings demonstrate that higher vancomycin doses in PwO are safely tolerated and do not result in short-term adverse effects on renal function. This study helps us better understand vancomycin pharmacotherapy in PwO, supporting the need for further research to refine dosing guidelines for these patients.

## Introduction

Obesity has emerged as a pressing public health concern worldwide, and its prevalence has continued to escalate in recent years. In the United States, the prevalence of obesity has reached alarming levels, with the Centers for Disease Control and Prevention (CDC) reporting that 42.4% of adults were obese in 2020, marking a significant increase from previous years [1]. Within this concerning landscape, obesity rates in the state of West Virginia have consistently ranked among the highest in the United States, with the CDC reporting a prevalence of obesity, defined as a BMI of 30 or higher, of 40% or greater in 2022 [2]. These statistics emphasize the pressing need for effective strategies to address the complex challenges posed by obesity.

Obesity not only predisposes individuals to health complications but also presents unique challenges when prescribing medications due to body composition and physiological changes. In patients with obesity (PwO), increased body fat and altered lean body mass result in an increased volume of distribution for lipophilic drugs, such as vancomycin. This necessitates higher doses to achieve serum concentrations comparable with those in non-obese individuals. Traditional dosing methods often lead to subtherapeutic drug levels in PwO, potentially resulting in treatment failure and the development of drug resistance [3]. Furthermore, the impact of obesity on renal clearance of vancomycin remains a critical area of investigation. Individuals with obesity often exhibit hyperfiltration, which can lead to faster clearance of drugs that are renally eliminated, thereby influencing dosing intervals [4]. Consequently, when considering the unique pharmacokinetic profile of PwO, there has been an ongoing debate regarding optimal vancomycin dosing regimens that maximize efficacy while minimizing risk.

There is considerable variation in the approaches to maximal dosing, frequency of administration, and initial loading doses for vancomycin, particularly when treating adults with obesity. The American Society of Health-System Pharmacists recommends considering a loading dose of 20-25 mg/kg to rapidly achieve therapeutic levels, especially in critically ill patients [5]. For maintenance dosing, some experts advocate for dosages as high as 15-20 mg/kg every 8-12 hours in PwO due to their altered pharmacokinetics [5,6]. Achieving and maintaining adequate trough concentration within the therapeutic range is also important to optimize therapeutic outcomes while minimizing toxicity. Vancomycin-associated nephrotoxicity represents a major potential adverse effect, with changes in renal function correlating with mortality and longer hospital stays [7].

Despite the increasing prevalence of obesity, the pharmacokinetics of vancomycin in PwO remain poorly understood. Therefore, our study aims to address one of the existing gaps by prospectively comparing two vancomycin loading dose regimens in PwO. By elucidating the most effective dosing strategy for PwO, this study attempts to optimize therapeutic outcomes and minimize vancomycin-associated adverse events.

## Materials and methods

Study design

This was a prospective, randomized, single-center, open-label, controlled clinical trial conducted from August 2016 to August 2021 at a regional referral center. Eligible patients met the following criteria: being 18 years of age or older, presenting to the Charleston Area Medical Center Memorial Hospital Emergency Department (Charleston, West Virginia, USA), weighing >100 kg and obese (with a BMI ≥30), and having an infection requiring both intravenous vancomycin (determined empirically) and hospital admission. Patients were excluded if they were on dialysis upon admission to the emergency department, had unstable renal function defined as a change of >0.5 mg/dL in serum creatinine (SCr) in patients with an SCr of <2 mg/dL or a 20% change in SCr in patients with an SCr of ≥2 mg/dL, or had an initial SCr of >2.5 mg/dL.

Written informed consent was obtained after potential candidates were identified and before vancomycin was ordered. Patients were randomized to one of two loading dose regimens for their initial dose of vancomycin using a computerized random number generator. All aspects of the study were approved by the Charleston Area Medical Center/West Virginia University-Charleston Institutional Review Board (IRB number: 15-210). This trial was registered with ClinicalTrials.gov under the registration number NCT02764359.

Vancomycin dosing regimens

The route of vancomycin administration for all participants was intravenous. Patients were randomized to receive a vancomycin loading dose of 20 mg/kg with a maximum dose of 2000 mg (group one) or a vancomycin loading dose of 20 mg/kg with a maximum dose of 4000 mg (group two). The hospital pharmacy department was informed of the patient’s enrollment and randomized the loading dose. Following administration of the vancomycin loading dose, vancomycin dosing was at the discretion of the attending physician, followed the hospital’s standard of care, and was monitored by the pharmacy department. In patients with serious, documented Gram-positive infections (e.g., endocarditis, meningitis, pneumonia, osteomyelitis), maintenance of a targeted steady-state trough concentration of 15-20 mg/L was attempted, while in patients with a lower risk of Gram-positive infections (e.g., pyelonephritis, skin/tissue structured infections), a target steady-state trough concentration of 10-15 mg/L was employed [8]. Goal trough levels followed the study hospital’s vancomycin guidelines.

Study outcomes

The primary endpoint was the serum vancomycin levels obtained after the loading dose (first vancomycin dose). Following administration of the loading dose, serum vancomycin levels were assessed at three time points, which comprised non-standard care. These measurements assessed differences in serum vancomycin levels and vancomycin pharmacokinetics between the two treatment regimens. The three assessments included post-load peak vancomycin level: drawn one hour after the loading dose administration (time window allowance for draw: 60-120 minutes for administration); 7.5-hour vancomycin level: drawn 7.5 hours (+/- 60 minutes) after the administration of the loading dose; and initial trough level: drawn 30 minutes before the second dose of vancomycin was scheduled (time window allowance for draw: 60 minutes on either side of the scheduled time for vancomycin dose as long as prior to the second vancomycin infusion).

The secondary endpoints included the incidence and type of adverse events following the administration of vancomycin treatment. Vancomycin flushing syndrome (VFS), characterized by an anaphylactoid reaction resulting from rapid infusion of vancomycin, was identified as a pruritic, erythematous rash affecting the face, neck, upper torso, and extremities. Nephrotoxicity was defined as a rise in SCr by 0.5 mg/dL, or 50% from baseline, in two consecutive measurements [9]. The occurrence of nephrotoxicity was evaluated within 48 hours following the vancomycin loading dose and 24 hours after the final vancomycin administration or hospital discharge. To provide a comprehensive assessment of renal function, creatinine clearance (CrCl) and estimated glomerular filtration rate (eGFR) were computed. CrCl was calculated utilizing the Cockcroft-Gault equation, which incorporates patient age, sex, total body weight (TBW), and SCr levels. Additionally, the following outcomes were compared: time needed to reach therapeutic vancomycin concentrations, intensive care unit (ICU) length of stay (LOS), hospital LOS, and in-hospital mortality between the two vancomycin loading dose regimens.

Data collection

All data were obtained prospectively to ensure a complete, valid data set for each patient. To better characterize our study groups, we obtained data for the variables of patient sex, weight, BMI, type of infection requiring vancomycin (pneumonia, sepsis, or skin/soft tissue), comorbidities of interest, presence of diabetes, and laboratory values for blood urea nitrogen (BUN), SCr, white blood count (WBC), lactate, and concomitant nephrotoxic medications. We defined nephrotoxic medications as agents that potentially alter renal hemodynamics and included acyclovir, aminoglycosides, angiotensin-converting enzyme inhibitors, angiotensin II receptor blockers, cephalosporins, contrast dyes, cyclosporine, diuretics, nonsteroidal anti-inflammatory drugs, tacrolimus, and vasopressors. Patients were stratified as being exposed to none, one, or two or more agents.

We also obtained vancomycin dosing information, including goal vancomycin trough level, loading vancomycin dose (mg/kg), total dose (mg), and administration time. Vancomycin maintenance dose (mg/kg and total), frequency of dosing, duration of vancomycin therapy (days and number of doses), and timing of the first therapeutic vancomycin through reaching the goal range were recorded. Loading and maintenance dose information was obtained from medication administration records located in participants' electronic health records. Once a patient was discharged from the hospital, they were considered to have completed the study.

Pharmacokinetic parameters, including the elimination rate constant and half-life, were assessed. The elimination rate constant was derived by calculating the natural logarithm of the ratio of two serum vancomycin concentrations and dividing by the time interval between the two measurements. The half-life was subsequently determined by dividing the natural logarithm of two by the elimination rate constant.

Statistical analysis

Our preliminary data indicated that 66 participants (33 per group) were required to have a 30% change in the primary outcome measure of initial trough level to be statistically significant when using two-tail Mann-Whitney U analysis (power = 0.80, p=0.05). This power analysis was performed using G*Power version 3.1.6 (Heinrich-Heine-Universität Düsseldorf, Germany). Patients were consented until 33 participants per study group were achieved.

Descriptive statistics were used to summarize the data. For continuous variables, if the data were normally distributed, results were expressed as mean ± standard deviation (SD). Normality was assessed using the Kolmogorov-Smirnov (KS) test. If the data were not normally distributed, we reported the median and range (min-max). Categorical variables were summarized as frequencies and percentages. Comparisons between groups were performed using the Student's t-test for normally distributed data and the Mann-Whitney U test for non-normally distributed data. For categorical variables, we used the Chi-square test or Fisher’s exact test, as appropriate. Initial trough vancomycin levels were stratified into subtherapeutic, therapeutic, or supratherapeutic categories based on patients’ goal trough and compared between study groups. For all comparisons, a p-value of <0.05 was considered statistically significant. Statistical analysis was performed on SAS version 9.4 (SAS Institute Inc., Cary, NC, USA).

## Results

Study participants

**Figure 1:**
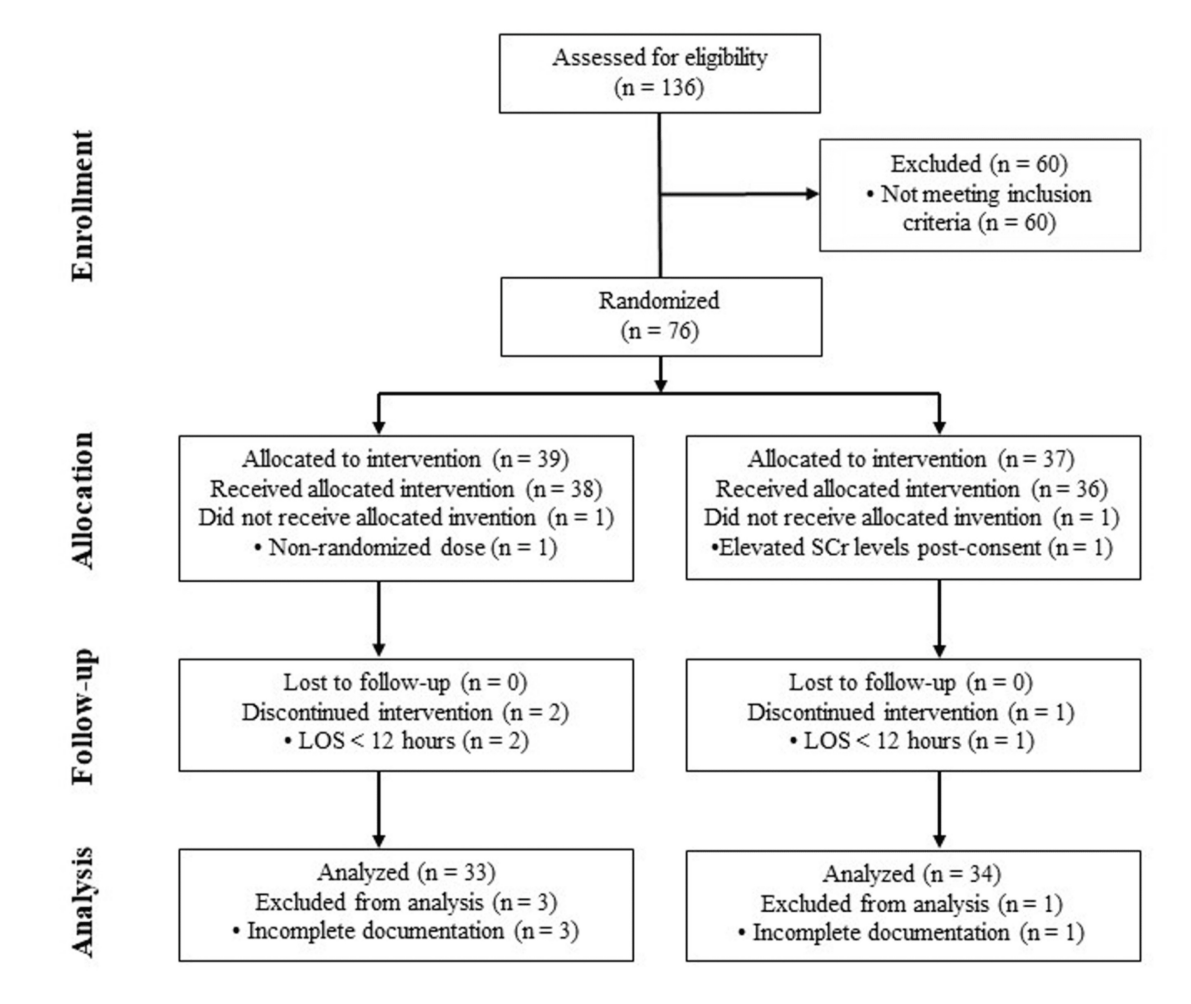
CONSORT flow diagram of the progress of patients through the study LOS: length of stay, SCr: serum creatinine, CONSORT: consolidated standards of reporting trials

A total of 136 participants were assessed for eligibility, with 60 excluded for not meeting inclusion criteria, leaving 76 participants who were randomized into two intervention groups. In the first group, 39 were allocated to the intervention, with 38 receiving it; one did not due to a non-randomized dose. In the second group, 37 were allocated, with 36 receiving the intervention; one did not due to elevated SCr levels post-consent. No participants were lost to follow-up in either group, but one from the first group and two from the second discontinued the intervention due to a LOS of less than 12 hours. For analysis, 33 participants from the first group were included, with three excluded due to incomplete documentation, while 34 from the second group were included, with one excluded due to incomplete documentation (Figure 1).

Participants' demographics and clinical characteristics

The mean BMI for the study participants was 45.8 ± 13.1 and ranged from 30.6 to 97.9. With the exception of age, there were no statistically significant differences in baseline demographics between the study groups (Table 1). No statistically significant differences in the rate of patients receiving nephrotoxic agents between the study groups were observed. The proportion of patients admitted to an ICU was comparable between study group one (21%) and study group two (15%, p=0.5387). No statistically significant disparities between the study groups were observed in comorbidities or infection types. No significant differences were noted for baseline laboratory values or kidney function surrogates.

**Table 1:** Summary of demographics and clinical characteristics Data are presented as means ± SD, medians (min-max), or numbers (%). Statistical comparisons were made using the t-test (t), Wilcoxon rank-sum (Z), Fisher’s exact test, or Chi-square test ( χ2) as appropriate. p<0.05 was considered statistically significant. BMI: body mass index, BUN: blood urea nitrogen, CrCl: creatinine clearance, ED: emergency department, eGFR: estimated glomerular filtration rate, ICU: intensive care unit, KS: Kolmogorov-Smirnov, SCr: serum creatinine

	Study group one (N=33)	Study group two (N=34)	KS value	Test value	p-value
Maximum vancomycin dose	2000 mg	4000 mg	-	-	-
Age	60.0 ± 12.0	53.5 ± 13.7	>0.15	t=2.05	0.0448
Height, cm	180 (157-206)	175 (155-195)	0.01	Z=1.09	0.2760
Body weight, kg	132 (104-257)	126 (104-215)	<0.01	Z=0.53	0.5983
BMI, kg/m^2^	42.3 (30.6-97.9)	41.5 (31.1-83.2)	<0.01	Z=-0.43	0.6698
Sex	-	-	-	χ^2^=1.37	0.2424
Male	23 (70%)	19 (56%)	-	-	-
Female	10 (30%)	15 (44%)	-	-	-
Race	-	-	-	-	1.0000
White	32 (97%)	32 (94%)	-	-	-
Black	1 (3%)	2 (6%)	-	-	-
Concomitant nephrotoxic medications	-	-	-	-	0.6996
None	21 (64%)	25 (74%)	-	-	-
One	7 (21%)	5 (15%)	-	-	-
Two or more	5 (15%)	4 (12%)	-	-	-
ICU admission	-	-	-	-	0.5387
Admitted	7 (21%)	5 (15%)	-	-	-
Not admitted	26 (79%)	29 (96%)	-	-	-
Comorbidities	-	-	-	-	0.9378
Diabetes mellitus	23 (70%)	19 (56%)	-	-	-
Chronic heart failure	8 (24%)	6 (18%)	-	-	-
Sepsis	7 (21%)	4 (12%)	-	-	-
Vancomycin indication	-	-	-	-	0.8569
Sepsis	18 (55%)	17 (50%)	-	-	-
Skin/soft tissue infection	20 (61%)	16 (47%)	-	-	-
Pneumonia	9 (27%)	10 (29%)	-	-	-
Other	1 (3%)	2 (6%)	-	-	-
ED admission labs	-	-	-	-	-
BUN, mg/dL	16.0 (10.0-42.0)	16.0 (8.0-53.0)	<0.01	Z=0.37	0.7109
WBC, 10^9^ cells/L	10.4 (2.9-27.3)	13.9 (5.0-25.3)	0.026	Z=-1.79	0.0739
Lactate, mmol/L	1.9 (1.0-4.0)	1.9 (1.1-7.4)	<0.01	Z=-0.19	0.8506
SCr, mg/dL	1.0 (0.5-1.6)	0.9 (0.5-2.0)	<0.01	Z=0.27	0.7864
CrCl, mL/min	156 (62-357)	169 (56-447)	0.045	Z=-0.08	0.9350
eGFR, mL/min/1.73m^2^	70.8 ± 22.7	69.9 ± 30.4	>0.15	t=0.13	0.8989

Vancomycin serum levels and pharmacokinetic data

Because all study participants weighed >100 kg, all patients in study group one received a 2000 mg loading dose. The median vancomycin loading dose was 15.2 mg/kg in study group one. The median loading dose in study group two was 2500 mg or 20.2 mg/kg (Table 2). Median days of vancomycin therapy did not differ between the study groups (p=0.14). Significant differences were observed in mean post-loading peak vancomycin levels, with study group two (4000 mg maximum dose) exhibiting higher peak serum vancomycin levels as compared with study group one (2000 mg maximum dose) (30.9 mg/mL vs. 23.9 mg/mL, p=0.0023). The 7.5-hour median vancomycin level was significantly higher in study group two as compared with study group one (17.1 mg/mL vs. 14.4 mg/mL, p=0.0150). The median initial trough level was also greater in study group two as compared with study group one (11.3 mg/mL vs. 9.1 mg/mL, p=0.0497). When initial trough levels were stratified based on goal troughs, the patients in the 4000 mg maximum group had a significantly higher rate of meeting therapeutic values (43%) as compared with the 2000 mg maximum study group (10%, p=0.03). The timing between vancomycin administration and measurement of serum vancomycin levels did not vary significantly between the two study groups (Table 2).

**Table 2:** Vancomycin treatments, serum levels, and pharmacokinetic data Data are presented as means ± SD, medians (min-max), or numbers (%). Statistical comparisons were made using the t-test (t), Wilcoxon rank-sum (Z), Fisher’s exact test, or Chi-square test ( χ2) as appropriate. p<0.05 was considered statistically significant. KS: Kolmogorov-Smirnov, q12h: every 12 hours, q24h: every 24 hours

	Study group one	N (%)	Study group two	N (%)	KS value	Test value	p-value
Vancomycin treatment	2000 mg maximum dose	-	4000 mg maximum dose	-	-	-	-
Loading dose, mg	2000 (2000-2000)	33 (100)	2500 (2000-4000)	34 (100)	<0.01	Z=-7.16	<0.0001
Loading dose, mg/kg	15.2 (7.8-19.2)	-	20.2 (18.0-30.0)	-	<0.01	Z=-6.87	<0.0001
Duration of therapy, days	3.0 (1.0-12.0)	-	2.5 (1.0-7.0)	-	<0.01	Z=1.08	0.2787
Dosing intervals	-	-	-	-	-	-	0.5345
q12h	27 (82%)	-	24 (71%)	-	-	-	-
q24h	4 (12%)	-	6 (18%)	-	-	-	-
Not recorded	2 (6%)	-	4 (12%)	-	-	-	-
Goal trough levels	-	-	-	-	-	χ2=1.53	0.4647
10-15, mg/kg	19 (58%)	33 (100)	16 (48%)	33 (97)	-	-	-
15-20, mg/kg	14 (42%)	-	17 (52%)	-	-	-	-
Post-load peak serum level, mg/L	23.9 ± 6.7	22 (67)	30.9 ± 7.9	25 (74)	>0.15	t=-3.23	0.0023
Time from infusion end to peak draw, min	98 (64-123)	-	88 (50-224)	-	0.04	Z=1.55	0.1274
7.5-hour serum level, mg/L	14.4 (5.2-22.1)	23 (70)	17.1 (11.2-45.0)	18 (53)	0.03	Z=2.43	0.0151
Time from infusion start to7.5-hour draw, min^-1^	447 (393-497)	-	451 (395-489)	-	0.05	Z=1.55	0.1210
Half-life at 7.5-hour level, hr	2.66 (1.40-11.62)	16 (48)	4.62 (2.20-12.65)	12 (35)	<0.01	Z=2.02	0.0434
Elimination constant at 7.5-hour trough level, hr^-1^	0.26 ± 0.12	16 (48)	0.17 ± 0.09	12 (35)	>0.15	t=2.31	0.0292
Initial trough level serum level, mg/L	9.1 (3.7-22.9)	21 (64)	11.3 (5.8-26.9)	23 (68)	<0.01	Z=-1.96	0.0497
Stratified according to goal trough	-	-	-	-	-	-	0.0300
Subtherapeutic	16 (76%)	-	10 (43%)	-	-	-	-
Therapeutic	2 (10%)	-	10 (43%)	-	-	-	-
Supratherapeutic	3 (14%)	-	3 (13%)	-	-	-	-
Time from infusion start to trough draw, min	733 (668-2088)	-	772 (612-2143)	-	<0.01	Z=-0.67	0.5030
Elimination constant at initial trough level, hr^-1^	0.49 ± 0.21	11 (31)	0.39 ± 0.18	15 (44)	>0.15	t=1.22	0.2359
Half-life at initial trough level, hr	1.50 (0.70-3.27)	11 (31)	1.9 (0.8-4.4)	15 (44)	0.03	Z=-1.25	0.2129
Time to Achieve Goal Trough, hr	16.6 (4.6-457.6)	24 (73)	26.2 (7.8-180.8)	19 (56)	<0.01	Z=1.06	0.2874

The elimination rate constant (ke) calculated at the 7.5-hour time point was significantly different between the study groups (p=0.0292), with the 2000 mg maximum study group having a mean ke of 0.26 ± 0.12 versus the 4000 mg maximum group, which had a mean ke of. 0.17 ± 0.09. Conversely, no statistically significant difference in elimination constants was noted at the initial trough. The median half-life calculated at the 7.5-hour trough was significantly longer in study group two (4.62 hours vs. 2.66 hours; p=0.0434). However, the difference in median half-life calculated at the initial trough was not statistically significant (p=0.2129). The time to achieve goal trough was also evaluated, and there was not a significant difference between the two study groups (p=0.2874).

Adverse events, kidney function, and patient outcomes

One case of VFS and four cases of nephrotoxicity occurred in the 4000 mg maximum dose study group (Table 3). All incidents of nephrotoxicity occurred within 48 hours of the loading dose. Overall, the rate of nephrotoxicity in the group that received the 4000 mg maximum dose was 12%, as compared to 0% in the group that received 2000 mg maximum (p=0.1139). Of the patients who exhibited nephrotoxicity, three patients were treated conservatively, while one required dialysis during hospitalization. Kidney function was stabilized in all patients prior to discharge. Hospital LOS and the ICU LOS did not significantly differ between groups (p=0.2534 and p=0.5053, respectively). Survival rates were also comparable between study groups; study group one had 100% survival, and study group two had a survival rate of 97% (Table 3).

**Table 3:** Adverse events and hospitalization Data are medians (min-max) or numbers (%). Statistical comparisons were made using Wilcoxon rank-sum (Z). p<0.05 was considered statistically significant. ICU: intensive care unit, KS: Kolmogorov-Smirnov, LOS: length of stay, VFS: vancomycin flushing syndrome

	Study group one (N=33)	Study group two (N=34)	KS value	Test value	p-value
Vancomycin treatment	2000 mg maximum dose	4000 mg maximum dose	-	-	-
Nephrotoxicity within 48 hrs of loading dose	0 (0%)	4 (12%)	-	-	0.1139
Nephrotoxicity within 24 hrs of final dose	0 (0%)	4 (12%)	-	-	0.1139
VFS	0 (0%)	1 (3%)	-	-	1.0000
In-hospital mortality	0 (0%)	1 (3%)	-	-	1.0000
Hospital LOS (days)	6.0 (0.0-59.0)	6.5 (2.0-24.0)	<0.010	Z=-1.14	0.2534
ICU LOS (days)	0.0 (0.0-22.0)	0.0 (0.0-16.0)	<0.010	Z=0.66	0.5053

Assessment of kidney function 24 and 48 hours after hospital arrival

Renal function 24 and 48 hours after hospital arrival was compared with baseline post-admission laboratory findings (Table 4). There were no significant differences in renal function between the two study groups.

**Table 4:** Assessment of kidney function 24 and 48 hours after hospital arrival Data are medians (min-max), number (%), and mean ± SD. Statistical comparisons were made using the t-test (t) or Wilcoxon rank-sum (Z). p<0.05 was considered statistically significant. CrCl values are mL/min, eGFR values are mL/min/1.73m^2, and BUN values are mg/dL. BUN: blood urea nitrogen, CrCl: creatinine clearance, KS: Kolmogorov-Smirnov, SCr: serum creatinine

	Study group one	N (%)	Study group two	N (%)	KS value	Test value	p-value
-	2000 mg maximum dose	-	4000 mg maximum dose	-	-	-	-
Δ CrCl, 24 hr	-6.6 ± 39.5	33 (100)	0.1 ± 42.4	34 (100)	0.105	t=-0.67	0.5022
Δ CrCl, 48 hr	0 .0 (-122.1-78.5)	29 (88)	4.0 (-123.4-89.5)	31 (91)	<0.010	Z=-0.15	0.8819
Δ eGFR, 24 hr	-1.3 ± 12.7	33 (100)	1.5 ± 15.4	34 (100)	0.135	t=-0.80	0.4276
Δ eGFR, 48 hr	0.0 (-35.4-51.0)	29 (88)	2.4 (-72.2-26.8)	31 (91)	<0.010	Z=0.21	0.8353
Δ SCr, 24 hr	0.0 (-0.3-0.5)	33 (100)	0.0 (-0.6-0.9)	34 (100)	<0.010	Z=0.58	0.5629
Δ SCr, 48 hr	0.0 (-0.8-0.9)	29 (88)	-0.1 (-0.5-1.7)	31 (91)	<0.010	Z=-0.19	0.8525
Δ BUN, 24 hr	-0.3 ± 4.8	33 (100)	-1.5 ± 4.6	34 (100)	0.149	t=1.02	0.3121
Δ BUN, 48 hr	-2.9 ± 8.4	29 (88)	-2.5 ± 9.6	31 (91)	>0.150	t=-0.21	0.8316

## Discussion

This study aimed to enhance the understanding of vancomycin dosing in PwO, a demographic often underrepresented in pharmacology studies. Our findings demonstrate that PwO administered a vancomycin dose of 20 mg/kg with a maximal dose of 4000 mg exhibited significantly higher post-load peak, 7.5-hour, and initial-trough vancomycin serum concentrations than patients administered a maximal dose of 2000 mg. Additionally, the extended half-life and altered elimination constant observed at the 7.5-hour measurement in patients given 20 mg/kg with a maximal dose of 4000 mg indicate prolonged retention and slower clearance of the drug. These findings suggest that higher doses can enhance effectiveness by rapidly achieving therapeutic drug levels in PwO. However, despite the difference in dosing, the time to reach goal trough levels did not differ between the study groups, suggesting a nuanced relationship between dose and the kinetics of vancomycin. These findings support previous research postulating that obesity can affect drug pharmacokinetics, necessitating dose adjustments to achieve therapeutic efficacy in PwO [10].

Literature on vancomycin dosing strategies in PwO is limited, and these individuals often receive subtherapeutic doses of vancomycin [3,11]. Achieving therapeutic serum levels of vancomycin in individuals with obesity can be challenging due to their increased volume of distribution, altered drug clearance, increased circulating proteins, and increased cardiac output and blood volume [12]. However, the high-dose vancomycin required to achieve target trough levels therapy in PwO may consequently have nephrotoxic effects [13]. In PwO, conventional vancomycin dosing starts with a loading dose of 20-25 mg/kg based on TBW and requires maintenance dosing at 15-20 mg/kg every 8-12 hours, with continual monitoring for renal response [5,6]. However, to our most current understanding, there has not been a defined maximum dose limit for this patient population. Our randomized study suggests that a 4000 mg maximal dose is effective and well-tolerated.

The potential for nephrotoxicity associated with higher vancomycin doses has been studied by others, with existing literature presenting a complex relationship. In 2019, Mei et al. performed a systematic review and meta-analysis and found that using higher loading doses of vancomycin was safe and achieved therapeutic concentrations without increased nephrotoxicity risk, contingent on rigorous monitoring [14]. However, their study focused on the clinical efficacy of using loading doses of vancomycin in the treatment of infections rather than exclusively analyzing PwO. Conversely, Lodise et al. identified an elevated risk of nephrotoxicity in patients with higher vancomycin exposure, particularly PwO exceeding 100 kg TBW, and advocated for meticulous dosing and monitoring to mitigate the risk of renal impairment [13]. Although we observed some incidences of nephrotoxicity among patients receiving a higher loading dose, it remains unclear whether the loading dose regimen contributed to these outcomes. However, pre-existing factors likely increased the risk in the patients with nephrotoxicity in this study. All four patients were septic at ED presentation, and three patients had a history of chronic kidney disease. Additionally, there were no significant differences in changes in renal function from baseline levels between our dosing strategies. This suggests that short-term renal function was not adversely affected, advocating for the potential safe use of higher vancomycin loading doses in PwO with appropriate monitoring. Patient-specific dosing and the utilization of advanced pharmacokinetic monitoring techniques may be required in patients with risk factors, including obesity, to maintain optimal kidney health. The absence of group-level differences does not exclude the possibility of individual variations in renal function.

Our findings demonstrate that allowing a higher vancomycin loading dose resulted in significantly higher trough vancomycin serum concentrations without leading to a significant change in LOS or mortality. This suggests the potential for optimizing dosing strategies to achieve therapeutic levels more rapidly in PwO. However, although the loading dose that each patient received was randomized, this study did not control for the influence of comorbid conditions. PwO often have comorbid conditions that can independently affect hospital LOS and mortality. Our findings support the importance of independently accounting for comorbid conditions, in addition to obesity, when performing individualized dosing and therapeutic drug monitoring.

Limitations were present in our study. First, the single-center design and the exclusion of patients with unstable renal function may limit the generalizability of our findings. Additionally, the study period’s overlap with the COVID-19 pandemic may have influenced patient demographics and hospital protocols, potentially contributing to greater-than-usual variations in the timing of blood draws by hospital personnel to assess vancomycin levels. Another limitation is that we did not analyze all possible confounders, including other concomitant medications, which may have influenced examined outcomes. Lastly, the study was limited to patient randomization for only the initial vancomycin dose under the supervision of the attending physician, with all subsequent vancomycin administration at the admitting physician’s discretion.

A major strength of this study is that it is the first to prospectively investigate the effects of a high vancomycin loading dose, up to 4000 mg, in a high-risk group of PwO, with 67.2% of enrolled participants meeting the definition of morbid obesity (BMI >40). All required treatment for serious infections with intravenous vancomycin. Furthermore, an additional trough level obtained at 7.5 hours after administration of the loading dose allowed for a consistent assessment of serum trough concentrations without variations in timing. This may provide novel insights to drive future research.

Due to heterogeneity in vancomycin dosing in PwO, further research is needed to refine dosing strategies. Multicenter trials, larger sample sizes, and a more diverse patient population could provide deeper insights into the optimal management of vancomycin therapy for this population. Additionally, longitudinal studies assessing long-term outcomes and nephrotoxicity rates are critical for developing an established and comprehensive dosing regimen. Continual monitoring of renal function biomarkers beyond the initial 48-hour period may further inform us regarding the effects on long-term kidney health and function. Lastly, the analysis of the area under the curve and minimum inhibitory concentration ratios could provide deeper insights into the optimal management of vancomycin therapy and lead to refined guidelines for patients who are morbidly obese.

## Conclusions

This prospective, randomized clinical trial contributes to the existing body of knowledge on vancomycin dosing for PwO. By directly comparing serum concentrations, renal function, and patient outcomes after administering a 4000 mg maximum loading dose versus a 2000 mg maximum loading dose, we found that higher vancomycin doses lead to higher vancomycin serum concentrations without immediate adverse impact on renal function. However, while higher doses achieved serum concentrations, we did not find improvement in examined clinical outcomes. These findings highlight the complexity of vancomycin dosing in PwO and underscore the necessity of individualized monitoring and dosing strategies.
